# Anticipating Surprises by Expanding a Risk Assessment's Possibility Space Into the Unknown With *C*–*K* Theory

**DOI:** 10.1111/risa.70406

**Published:** 2026-10-29

**Authors:** James Derbyshire

**Affiliations:** ^1^ Chester Business School University of Chester, Queen's Park Campus Chester UK

**Keywords:** *C*–*K* theory, risk assessment, surprises, unknowledge

## Abstract

What should a risk assessor do if she suspects the possibilities derivable from presently available knowledge are incomplete? Risk analysts often encounter this problem when assessing risk in open systems that produce novel outcomes leading to surprises. The solution is to expand the possibility space into the unknown. Yet the novel possibilities then included cannot be mere figments of the imagination; they must be tethered to presently available knowledge to be credible. This may seem paradoxical: to go beyond presently available knowledge by assessing novel possibilities, which, to be credible, must be grounded in presently available knowledge. Yet it is not quite as contradictory as it may first seem. *C*–*K* Theory (CKT) was developed to explain how innovators overcome a similar problem when designing a new product. An innovation involves creating something that has novel features. Some of its components may, therefore, not yet exist, and there may even be uncertainty about whether they are technically feasible. This lack of knowledge makes it difficult to determine the innovation's possibility space—a difficulty similar to that faced when assessing risks in open systems. CKT describes how innovators overcome this problem by iteratively moving between a knowledge space comprising established facts and decidable propositions and a conceptual space representing novel, as‐yet‐undecidable propositions. As illustrated herein using the cases of the credit crunch and the 9/11 terrorist attack, CKT can help anticipate surprises by generating, expanding, and integrating the possibility space within the extended (*C*, *U*) risk‐assessment framework.

## 1 Introduction

On November 5, 2008—the day on which British people annually celebrate the foiling of Guy Fawkes’ plot to blow up the Houses of Parliament—a blow‐up of an entirely different type was the topic of discussion. It was almost 2 months after the Lehman Brothers bankruptcy, which precipitated a descent into the abyss for Western economies (Chorafas 2013). Queen Elizabeth II was inaugurating a new building at the London School of Economics. When discussing the financial blow‐up^1^—which, unlike that planned by Guy Fawkes, went unnoticed until it was too late—with the institution's highly distinguished professors, the Queen asked why nobody had noticed its coming (Dow et al. 2009). The professors were surprised by the question, so only later did two of them—Professors Tim Besley and Peter Hennessy—write an open letter to the Queen suggesting that the financial crisis was “… principally a failure of the collective imagination of many bright people…to understand the risks to the system as a whole” (Chorafas 2013, 15).

Yet, in a much less vaunted open letter written in response to that of Besley and Hennessy, Dow et al. (2009) implied that this explanation was somewhat glib because it glossed over the reasons for the failure of collective imagination. Among them is the insufficient attention economists pay to historical precedents, which stems in part from mainstream economics’ excessive emphasis on mathematical modeling (Dow et al. 2009). Indeed, as discussed later, extreme events like the credit crunch may be novel, but they often rhyme with historical precedents, even if they never exactly repeat them. For that reason, anticipating them is not entirely dependent on making a lucky stab in the absolute dark. With the right tools and methods, we can use history's rhyme scheme to anticipate the rhyming couplet at the end of the next line^2^ (i.e., the next event of a given type).

Gilain et al. (2023) ask what a decision‐maker should do if she suspects that the possibilities derivable from presently available knowledge are incomplete and could be augmented by presently unknown alternatives that are difficult to imagine. That question is particularly salient for risk assessment in open systems (e.g., the financial system), in which novel outcomes are expected (Cox 2020). In open systems, bounding the possibility space based exclusively on presently available knowledge leaves the door open to surprises (Derbyshire and Aven 2025). Anticipating surprises in such systems, therefore, requires risk assessors to look beyond presently available knowledge. They must expand the possibility space into the unknown by generating and considering novel possibilities. Yet, the novel possibilities then considered cannot be mere works of fiction or figments of the imagination. To be credible, they must be tethered to or grounded in evidence in some way.

This may initially seem like a paradox: to go beyond presently available knowledge to consider novel possibilities, which must be tethered to or grounded in the evidence provided by that knowledge. Yet, as contradictory as this may seem at first, new models of generative rationality enable just that. By partitioning, decomposing, and recombining established facts and decidable propositions, such models generate unknowns from knowns, then transform the novel possibilities they represent into new established facts and decidable propositions. This expands the possibility space into the unknown in a way that is grounded in but not restricted by presently available knowledge.

This article draws on Le Masson et al. (2019), Gilain et al. (2023), and Hatchuel and Weil (2003, 2009) to consider the implications of using one such model of generative rationality in risk assessment: *C*–*K* theory (CKT). This model of generative rationality was developed in innovation and design studies (Hatchuel and Weil 2003, 2009). Innovation involves creating something that has novel features. Some vital components of an innovation may not yet exist, and there may be uncertainty about whether they are even technically feasible. This lack of knowledge makes it difficult to determine the possibility space. Assessing risks in an open system presents a similar difficulty (Cox 2020).

CKT describes how innovators overcome this problem. It can help operationalize the (*C*, *U*) risk‐assessment framework, which was recently extended to explicitly account for unknowns (Derbyshire and Aven 2025). It helps to generate and integrate the expanded knowledge landscape from which that framework's possibility space is derived. Expanding the knowledge landscape narrows the gap between the full range of possibilities that could occur over infinite time and those known to the risk assessors at the time of assessment. In other words, it narrows the “knowledge gap” highlighted within the (*C*, *U*) risk‐assessment framework (Derbyshire and Aven 2025, 2026). Narrowing this knowledge gap reduces susceptibility to surprises.

The plan for this article is as follows. Section 2 introduces CKT. Section 3 illustrates the use of CKT in a risk context using two cases: the credit crunch and the 9/11 terrorist attack. Section 4 discusses how CKT helps operationalize the extended (*C*, *U*) risk‐assessment framework. Section 5 discusses a few other approaches for expanding a possibility space into the unknown. Section 6 makes some concluding remarks.

## 2 A Brief Synopsis of CKT

Innovation involves designing something that does not presently exist in its entirety, with aspects of it therefore subject to uncertainty and unknowns (Derbyshire and Giovannetti 2017). For example, some components of an innovation may not yet exist, and it may be uncertain whether they are even feasible. This lack of knowledge makes it difficult to determine the innovation's possibility space and search it for novel possibilities. In this section, we draw on Hatchuel and Weil (2003, 2009) to outline and discuss CKT—a theory describing how innovators overcome this problem. This discussion is also informed by Gilain et al. (2023) and Le Masson et al. (2019), who elaborate on generative rationality by building on the foundations laid by Hatchuel and Weil (2003, 2009). Generative rationality is markedly different from the optimization‐based rationality of traditional risk analysis and decision‐making methods.

In CKT, the possibility space—or “design space”—is composed of two lower‐level spaces: (1) a conceptual space (*C*‐space), and (2) a knowledge space (*K*‐space). *C*‐space comprises novel propositions—that is, new ideas, potential objects, and broad specifications—that are initially of unknown validity (i.e., they are undecidable^3^) based on what is then presently available knowledge. *K*‐space comprises established facts and decidable propositions that have already been proven or falsified. The novel concepts in *C*‐space are all based on the general proposition that: “There exists some object *x*, for which a group of properties $P_{1}$, $P_{2}$, …, $P_{k}$ are true in *K*” (Hatchuel and Weil 2009, 182). These novel concepts, which are known as *C*‐sets, are partially unknown objects (Hatchuel and Weil 2009). The process of innovation involves transforming a *C*‐set into a *K*‐set—that is, transforming a set of novel concepts in *C*‐space into a set of established facts and decidable propositions in *K*‐space.

For example, as Hatchuel and Weil (2009, 184–185) elaborate, the designer of an innovation might start with a decidable statement based on established facts, such as “there exists an *x* with *a* set of attributes $A_{0}$.” The designer then changes the initial set of attributes $A_{0}$ into $A_{i}$ by adding or subtracting attributes, and by introducing some design parameters, $D_{i}$. In this way, a new proposition $C_{i}$ is made: “there exists an *x* with a set of attributes $A_{i}$, which can be made with a set of parameters $D_{i}$.” This results in three possibilities for the logical status of $C_{i}$ in *K*‐space (Hatchuel and Weil 2009, 185):

1. $C_{i}$ is false in *K*‐space and the designer must again change some of the $A_{i}$ *s* or the $D_{i}$ s to find a $C_{i}$ that is not false;
2. $C_{i}$ is true in *K*‐space and ($D_{i}$, $A_{i}$) is therefore an example of a candidate “solution” for *x*, which is referred to in CKT as a “conjunction for *x*”;
3. $C_{i}$ is presently undecidable and so remains neither true nor false in *K*‐space; it, therefore, requires further development, consideration, and experimentation as the design process continues.

In the first and second cases, new propositions have been added to *K*‐space; in the third case, a new concept has been added to *C*‐space. By proceeding in this way, the design process simultaneously extends *C*‐space and *K*‐space into an expanded possibility space. A key feature of CKT is therefore its “power of expansion” (Hatchuel and Weil 2009, 185). This power of expansion means CKT is highly relevant to risk assessment, a key requirement of which is to expand the possibility space into the unknown to capture novel possibilities that might otherwise be surprises (Derbyshire and Aven 2025).

In CKT, *C*‐space and *K*‐space are simultaneously expanded into a broader space of possibilities through iterative revisions to the identity of considered “objects” (Hatchuel and Weil 2009). Hatchuel and Weil's (2009) simple example is of a *K*‐space comprising propositions about a collection of objects *O*, which all have the attribute $A_{0}$ that is described by the proposition “All known car tyres are made of rubber.” $A_{0}$ (i.e., being made of rubber) is therefore a partial element of the identity of the object *O*. This proposition, which accommodates novel possibilities due to its openness (i.e., “all *known* car tyres …” and not “all car tyres …”), allows for a new proposition *Q* in *C*‐space, such as: “There exists an *O* that does not have the attribute $A_{0}$,” meaning that there can be a car tyre that is not made of rubber. *Q* therefore revises the attributes of *O* as presently conceived in *K*‐space. In this way, CKT captures the emergence of novel possibilities that expand the possibility space.

The designer of an innovation expands the possibility space by navigating back and forth between *C*‐space and *K*‐space via four “design operators” (Hatchuel and Weil 2003, 2009). This can be illustrated by considering the innovation, before any such car yet existed, of a self‐driving car that is capable of driving in cities. The four design operators and their roles in the expansion process are explained below (Hatchuel and Weil 2003, 2009):

1. Concept expansion within *C*‐space (*C *→ *C*): A novel concept is initially generated in *C*‐space through disjunction from *K*‐space (*K *→ *C*). This involves “partitioning” (e.g., adding new attributes to) an established fact and decidable proposition in the form of a knowledge element, or combination of knowledge elements, already residing in *K*‐space. For example, in the early development of autonomous vehicles, “a self‐driving car” might have been a known and proven object already in *K*‐space, which might then have been disjunctioned into the concept of “a self‐driving car capable of driving in cities,” which, at that time, would have been a novel and undecidable proposition. This novel proposition could have been further refined through increasingly specific partitions in *C*‐space (*C *→ *C*), creating a branching array of novel concepts tethered to established knowledge elements already residing in *K*‐space (*K *→ *C*).
2. Concept to knowledge (*C *→ *K*): Partitioning a concept through (*C *→ *C*) triggers the search for and generation of new knowledge (*C *→ *K*) that tests its feasibility. For example, testing the concept of “a self‐driving car capable of driving in cities” when no such car yet exists might have involved evaluating available sensor technology, considering the implications of liability laws, and examining the details of the autonomous systems used to guide similar technologies. Conducting these investigations will expand *K*‐space by adding new knowledge elements to it from *C*‐space (*C *→ *K*). New facts will be established in *K*‐space, and new propositions will be made decidable within it.
3. Knowledge to Concept (*K *→ *C*): The new knowledge elements added to *K*‐space through (*C *→ *K*) then influence *C*‐space through (*K *→ *C*). They act to circumscribe and reshape the development of the novel concept that gave rise to their generation in the first place. For example, if these new knowledge elements show that existing sensor technology is sufficient to enable self‐driving cars to operate in cities with low risk, then this newly generated knowledge would loosen the bounds of possibility and allow further refinement and partitioning of this novel concept in *C*‐space (*C *→ *C*), leading to a still more specific concept. The branching array of possibilities is thereby extended further. Conversely, if the knowledge generated through (*C *→ *K*) indicates that existing sensor technology only enables self‐driving cars to operate safely in cities under very restrictive conditions, then this will circumscribe the novel concept's further partitioning in *C*‐space (*C *→ *C*).
4. Knowledge expansion within *K*‐space (*K *→ *K*): Presently available knowledge elements are combined, recombined, restructured, and extended to generate new knowledge elements without necessarily referencing any new concept under consideration. For example, established facts and decidable propositions about sensors and traffic regulations might be combined to generate a new fact that affects the technical feasibility of producing a safe self‐driving car of any type.

Through these four design operators (*C *→ *C*, *C *→ *K*, *K *→ *C*, *K *→ *K*), a novel concept is generated that is initially undecidable. It then undergoes an iterative learning loop that makes it more concrete and specific, tests its feasibility, generates new knowledge elements about it, and reshapes it as a concept. As illustrated in Figure 1, this involves moving from knowledge of established facts and decidable propositions to new concepts that are initially undecidable given the knowledge then presently available. Through testing and experimentation of one form or another,^4^ these novel concepts are made decidable, and their status is transformed into that of an established knowledge element in *K*‐space. Through this back‐and‐forth iterative expansion, *C*‐space and *K*‐space are simultaneously expanded and integrated into a broader possibility space. In the next section, we apply CKT to risk assessment to consider how it might have aided anticipation of the credit crunch and the 9/11 terrorist attack.

**FIGURE 1:**
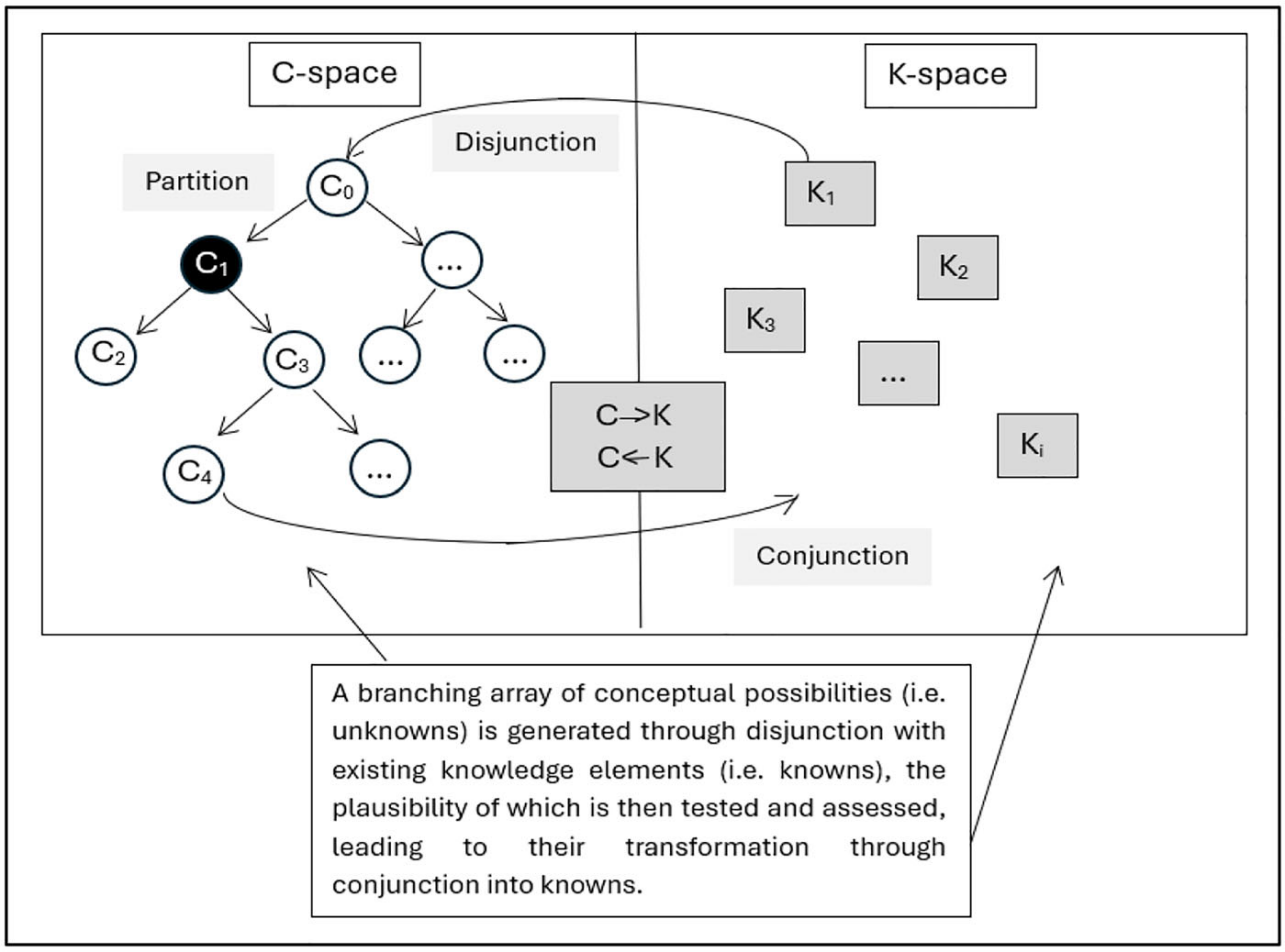
The process of simultaneously expanding and integrating *C*‐space and *K*‐space in *C*–*K* design theory. Adapted from Hatchuel and Weil (2009, 188).

## 3 Using CKT to Expand a Possibility Space Into the Unknown: Anticipating the Credit Crunch and 9/11

### 3.1 Anticipating the Credit Crunch

The credit crunch threatened the viability of major financial institutions and led to the collapse of one of the world's largest investment banks, Lehman Brothers. It was the worst financial crisis in nearly a century. Yet, the Nobel Prize‐winning economist Stiglitz (2010, 2) has suggested it was just another credit crisis resulting from a real estate bubble, which is “a dime a dozen” in history. It did, however, also have some novel aspects, such as the proliferation of complex financial instruments in the years leading up to it (Derbyshire 2023). These familiar and novel elements combined to create the credit crunch, which was both similar to and distinct from other financial crises (Claessens et al. 2010). Indeed, Claessens and Kose (2013) explicitly refer to financial crises as typically multifaceted yet sharing common elements.

As Mark Twain is claimed to have said then (Schoemaker 2020), history does not repeat itself, but it does rhyme. Similar events recur over time, but with their antecedents shuffled into a slightly different order and their leitmotifs complemented by a novel plot twist or two. Because financial crises exhibit both commonalities and novelties in this way, CKT can help in uncovering the rhyme scheme of their historical precedents, aiding anticipation of the rhyming couplet that is the next financial crash.

Claessens et al. (2010) describe four similarities between the credit crunch and earlier financial crises. First, in the years preceding the credit crunch, asset prices rose rapidly. Second, there were credit booms in several key economies. Third, there was a dramatic increase in a variety of marginal loans in mortgage markets across key economies. Fourth, regulators failed to keep up with these developments. These factors resonate strongly with historical precedents (Claessens et al. 2010). They are the established facts and decidable propositions that comprise the *K*‐space of an initial possibility space primed for expansion using CKT.

Now consider the *C*‐space. Claessens et al. (2010) also highlight four important differences between the credit crunch and previous financial crises. First, in the years preceding the crisis, complex financial instruments had become pervasive, increasing opacity within the financial system. Second, and relatedly, the interconnectedness of global financial markets increased markedly. This created the conditions for second‐order effects, which enable the spread of a contagion and generate positive feedback by amplifying initially small changes, leading to a buildup of systemic risk (Logan et al. 2026). Third, financial institutions’ leverage accelerated sharply. Fourth, the household sector became increasingly indebted.

Indeed, the economist Professor Steve Keen—who, unlike Besley and Hennessy, *did* anticipate the credit crunch—attributes the steep rise in private and household debt to a central role in the credit crunch (Keen 2009). As Keen (2009) states, private debt is largely ignored in conventional macroeconomics, whereas it is emphasized in less conventional Minskyan economics. Minsky (1977) highlighted the inherent tendency of financial markets towards instability and famously coined the term “Minsky moment” to describe the point at which all future expectations are collapsed into the present, causing a crash.

Minsky (1977) has done much to uncover the rhyme scheme of historical financial crashes. It goes something like this: during the growth phase of a financial bubble, investors expect asset values to continue rising indefinitely. They therefore borrow heavily to fund investments. However, when asset values inevitably stop increasing, this expectation for indefinite growth is shattered. Investors and financial institutions suddenly realize that the accumulated debt cannot be paid off by selling the inflated assets, as those assets are declining in value. Changed future expectations are then collapsed into the present moment, causing a sudden shift in sentiment, which leads to a financial crash.

And so it was with the credit crunch; only this time the proliferation of complex financial instruments made it even more difficult to assess changes in asset values and the robustness of bank balance sheets as expectations shifted. This proliferation of complex financial instruments (e.g., collateralized debt obligations) created a tangled and opaque web of debt in the financial system, increasing systemic risk (Brunnermeier 2009). The resulting opacity led to a collapse in trust and an unwillingness among banks to lend to each other (Derbyshire 2023).

Considering how CKT might have aided anticipation of this outcome: in a hypothetical central bank risk assessment before the credit crunch, the initial *K*‐space could have been populated with factors common to financial crises, such as those described by Minsky (1977) and in Claessens et al. (2010). Factors such as a rapid increase in asset prices, followed by a credit boom. Recall Hatchuel and Weil's (2009) simple example of a *K*‐space comprising propositions about a collection of objects *O*, which all have the attribute $A_{0}$—for example, “all known car tyres are made of rubber.” In the case of financial crises, we might consider the collection of “objects” *O* to be financial crises, which all initially have a common set of attributes $A_{0}$ in *K*‐space: “All known financial crises are characterised by a rapid increase in asset prices, followed by a credit boom.” This was a decidable proposition in *K*‐space.

Monitoring these established facts and their evolving relationships (e.g., tracking asset price increases and the rising indebtedness of banks and households) might, in itself, have provided an early warning of an impending crash. This would have expanded *K*‐space (*K *→ *K*) by making the possibility of a new financial crisis concrete. But what novel form might it take on this occasion? What would be the rhyming couplet at the end of the next line? Considering this matter would require a disjunction from the presently available knowledge in *K*‐space into *C*‐space (*K *→ *C*).

The established facts residing within *K*‐space and the proposition to which they had given initial rise ($A_{0}$) might have been disjunctioned into a novel possibility in *C*‐space (*K *→ *C*), leading to a new proposition. This might have been stimulated by considering the nature of modern financial instruments, such as a collateralized debt obligation, which packages debt of different types and qualities and assigns the package an aggregated risk that is something like an average (e.g., a so‐called triple A rating).

Expanding on and making more specific the initial proposition $A_{0}$, the novel proposition leading to a disjunction into *C*‐space (*K *→ *C*) might have been “There exists an *O* (i.e., a type of financial crisis) that is characterised by a rapid increase in asset prices, followed by a credit boom, *accompanied by the proliferation of complex financial instruments*.” The emphasized part of this new proposition represents a more specific partition of the decidable proposition already in *K*‐space ($A_{0}$). Now consider the further partition of this novel concept in *C*‐space (*C *→ *C*). Imagine that consideration had been given to the effect of the resulting opacity on banks’ ability to assess the viability of other banks to which they were lending.

For example, Stiglitz (2010) describes how, once the credit crunch was set in motion, banks began asking: As we cannot accurately value the assets on our own balance sheet, how can we possibly do so for other banks? This might have led to a further partition within *C*‐space (*C *→ *C*), perhaps based on the proposition “There exists an *O* (i.e., a type of financial crisis) that is characterised by a rapid increase in asset prices, followed by a credit boom, accompanied by the proliferation of complex financial instruments, *and leading to distrust spreading as a contagion through the banking system*.” The new specificity emphasized in this still more refined novel proposition captures what ultimately led to the proximate cause of the credit crunch: the contagious spread of distrust through the banking system (Derbyshire 2023). Its preceding clauses capture the more distal, root, or underlying causal mechanisms, which were a combination of common and novel factors.

This final partition is a still more specific refinement of the original proposition in *K*‐space. It might have triggered testing and further investigation to assess its plausibility, leading to the generation of new knowledge elements and their transfer into *K*‐space (*C *→ *K*). This brings the process of expansion full circle and illustrates how *K*‐space and *C*‐space are simultaneously expanded into an enlarged and integrated possibility space that incorporates a broader range of possibilities than those based on the knowledge then presently available at the beginning of the process.

Of course, this description of how a CKT‐informed approach might have helped anticipate the credit crunch is speculative, oversimplified in several ways, and undoubtedly benefits greatly from hindsight. Describing retrospectively how such events *may* have been anticipated is very different from identifying them as plausible possibilities prospectively. Derbyshire and Aven (2026) characterize the latter as searching for a straw in a haystack (Snowden 2024). When time's arrow winnows down the possibilities and separates that one important straw from all the others that appear just as plausibly to be the potential future, everybody can see that it should have been the focus of attention all along. But when it is entangled within the haystack, identifying it as such is fiendishly difficult.

The process of expansion we have described would no doubt have led to several branching arrays of novel possibilities in *C*‐space. Deciding which possibilities among them were plausible enough to be considered “knowledge” worthy of transfer into *K*‐space would be very difficult. Relatedly, it would also be very difficult to decide which was sufficiently plausible to warrant further risk analysis that would make the potential consequences of the established facts and propositions within *K*‐space more concrete. Nevertheless, the example provides an inkling into how CKT might have been used to expand the possibility space, perhaps increasing the likelihood of anticipating the credit crunch. We now turn, more briefly, to a second example that clears the ground for our later discussion of CKT's role in operationalizing the extended (*C*, *U*) risk‐assessment framework.

### 3.2 Anticipating the 9/11 Terrorist Attack

Just as the credit crunch was in Besley and Hennessy's open letter to the Queen, the failure to anticipate the 9/11 terrorist attack has been attributed to a collective failure of imagination (Weick 2005). Derbyshire and Aven (2026) use the 9/11 case to discuss how to stimulate collective imagination through distributed sensemaking. Below, we draw on the 9/11 case to provide a second, albeit somewhat briefer, illustration of how CKT might have aided anticipation in a real risk context, complementing the one on the credit crunch above. This further clears the ground for the discussion in the next section, which expands on Derbyshire and Aven (2026) by providing more details on how to operationalize the extended (*C*, *U*) risk‐assessment framework.

What makes the case of 9/11 salient to the present discussion is that all the knowledge necessary to anticipate the attack was known to one government agency or another. There was a failure to integrate the dispersed knowledge elements into a complete knowledge landscape that represents all presently available knowledge (Derbyshire and Aven 2026). This meant that an expanded possibility space could not be derived by generating novel possibilities through the juxtaposition of the alternative “frames of reference” (Schum 1994, 228) represented by the dispersed knowledge elements (Derbyshire and Aven 2026).

How might CKT have aided matters in this case? Once these dispersed knowledge elements are integrated—for example, by using the distributed sensemaking approach in Derbyshire and Aven (2026)—the matter then becomes one of expanding the possibility space by generating novel possibilities from a fully populated *K*‐space in which all presently available knowledge elements are represented. As described in Derbyshire and Aven (2026), two of these knowledge elements were “suicide attack” and “hijacking.” In a hypothetical CKT‐informed risk assessment, these two knowledge elements might have been associated with two decidable propositions in *K*‐space: (1) “There exists a type of terrorist attack in which the terrorist commits suicide” and (2) “There exists a type of terrorist attack in which an aeroplane is hijacked.” These propositions are established facts based on many historical precedents.

These established facts and decidable propositions might have been combined in *K*‐space (*K *→ *K*), perhaps yielding a disjunction into *C*‐space (*K *→ *C*), which, in turn, led to several further partitions within *C*‐space (*C *→ *C*). Through these, this expansion might have led to a novel concept along the lines of “There exists a type of suicide attack in which an aeroplane is hijacked and used as a missile.” In other words, a “suicide hijacking” (Derbyshire and Aven 2026). Testing its plausibility through various means might have led to the acceptance of this proposition and the possibility it represents, thereby transferring it into *K*‐space (*C *→ *K*). For example, the FBI knew before the attacks that terrorists were receiving pilot training at flight schools in the United States (Kroenig and Stowsky 2006, Footnote 44). Considering this knowledge element alongside other established facts and propositions within a comprehensive, integrated *K*‐space might have suggested that this novel possibility was highly plausible.

In this way, *C*‐space and *K*‐space might have been simultaneously expanded, increasing the chance of juxtaposing the established facts and decidable propositions into novel possibilities, among them being the possibility of a suicide hijacking in which airplanes are used as missiles. Derbyshire and Aven (2026) discuss how this possibility fell between the cracks of individual judgments and alternative frames of reference. A CKT‐informed approach might have made this eventuality less likely. Of course, the same caveats regarding the event's anticipation being far easier said retrospectively than done prospectively apply as in the case of the credit crunch.

## 4 Narrowing the Knowledge Gap and Anticipating Surprises: The Role of CKT in Operationalizing the Extended (*C*, *U*) Risk‐Assessment Framework

The extended (*C*, *U*) risk‐assessment framework (Derbyshire and Aven 2025) is designed to account for all types of uncertainty across all contexts. It helps anticipate what might otherwise be surprises by ensuring that a risk assessment does not overemphasize what is known about a focal risk matter at the expense of what is unknown. It recognizes that uncertainty and surprises arise from the absence of knowledge, which the economist Shackle (1955, 1961) called “unknowledge” (Derbyshire and Aven 2025).

The framework captures uncertainties of all types by conceptualizing risk in the broadest possible terms as the two‐dimensional combination:

(1) $$\left(C,U\right),$$

where *C* is the full range of potential consequences of a considered event or outcome, and *U* is the associated uncertainty (i.e., what will *C* be?). The specific risk from the event or outcome is then characterized in a risk assessment by five components that are considered together:

(2) $$\left(C\prime ,Q,K,UK,T\right),$$

where *C*′ is the set of consequences (e.g., the number of fatalities), *Q* is an appropriate measure or description of uncertainty, and *K* and UK are, respectively, knowledge and unknowledge. *K* represents presently available knowledge, and UK represents presently unavailable knowledge—or unknowledge—about *C*′ and *Q*. Unknowledge is therefore knowledge that would be relevant to the risk assessment if it were available but which is not presently available. Together, *K* and UK constitute the knowledge landscape for the risk assessment. The knowledge landscape (*K* and UK)―and, concomitantly, the set of consequences (*C*′) and their uncertainty (*Q*)―change over time (*T*).

Over time, new developments generate (and therefore augment), alter, or render obsolete (and therefore delete) the knowledge elements comprising the knowledge landscape (*K* and UK). As shown in Figure 2, this updates the knowledge landscape and, therefore, the set of events, outcomes, and consequences (*C*′), and their uncertainty (*Q*), which together make up the risk assessment's possibility space. Uncertainty (*Q*) reflects the “strength of knowledge”—or, alternatively, the “evidential weight” (Derbyshire et al. 2023)—that is represented on the knowledge landscape underpinning the assessment of the focal event or outcome and its consequences.

**FIGURE 2:**
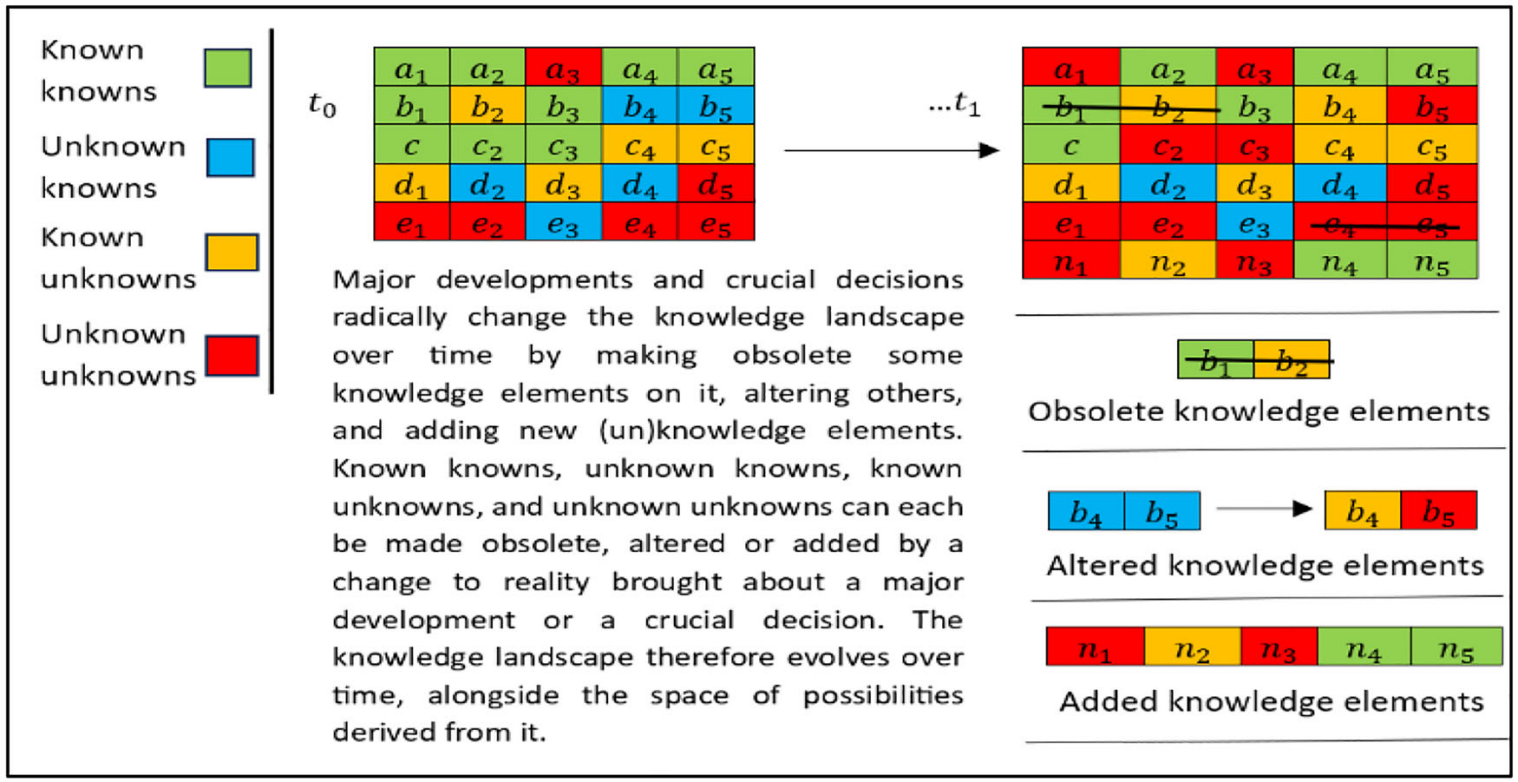
A knowledge landscape as represented in the extended (*C*, *U*) risk‐assessment framework. Adapted from Derbyshire and Aven (2025, 1203).

As shown in Figure 3, strength of knowledge, or evidential weight, is evaluated by considering the relative extent (on the knowledge landscape) of known and unknown knowledge elements about the occurrence and consequences of the focal event or outcome. A notable feature of this framework is therefore its ability to capture, clearly represent, and communicate situations of low knowledge strength (or low evidential weight) and high uncertainty. This ability is vital to maintaining the credibility and integrity of a risk assessment (Thekdi and Aven 2024).

**FIGURE 3:**
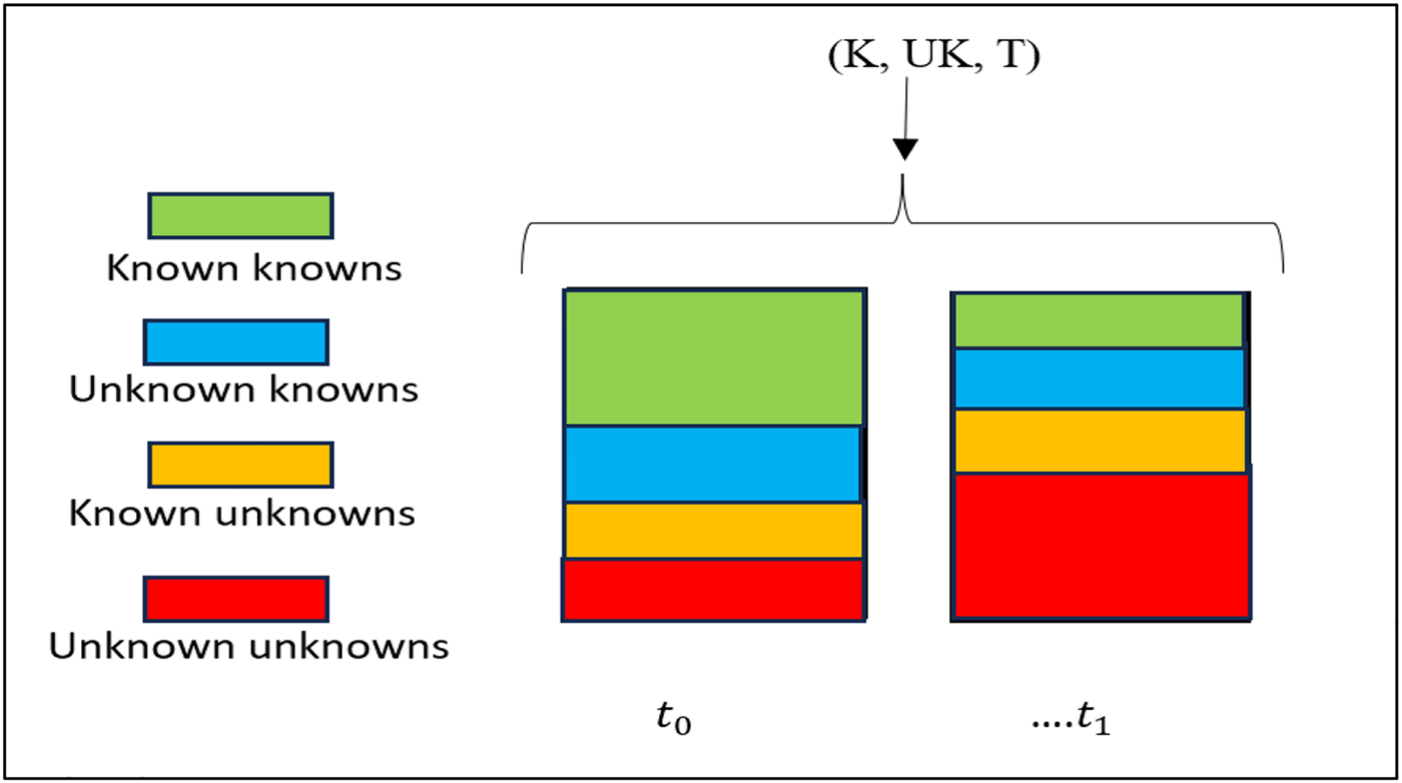
Representing uncertainty by evaluating the strength of knowledge underpinning the assessment of a considered event or outcome and its consequences. Adapted from Derbyshire and Aven (2025, 1205).

In this framework, surprises result from the knowledge gap between *C*′ in (2) and *C* in (1). Specifically, *C*′ in (2) represents the set of events or outcomes and associated consequences known to the risk assessor at the time of the assessment, as derived from the knowledge landscape available at that time, whereas *C* in (1) represents all the potential events or outcomes and associated consequences that could occur over infinite time, regardless of whether they are derivable from the knowledge landscape available at the time of the risk assessment—and, therefore, irrespective of whether they are known to the risk assessors at that time of assessment.

*C* in (1) essentially represents a hypothetical possibility space based on an absolutely complete knowledge landscape from which all possibilities over infinite time are derivable, meaning the knowledge gap has been entirely closed. However, such a possibility space can never be constructed because it is impossible to entirely close the knowledge gap. A knowledge landscape can contain all presently available knowledge, but it can never contain all the knowledge relevant to a focal matter as revealed over the fullness of time. There will always be matters that subsequently transpire to have been important that were not included in the knowledge landscape from which the possibility space is derived. Even if it were somehow possible to close the knowledge gap entirely at a single point in time, it would not remain closed for long. As the world is constantly changing (especially in an open system), an absolutely complete knowledge landscape, if it were ever possible to construct one, would be that way for the most solitary of present moments in the infinite flow of time (Derbyshire 2026).

Notwithstanding this, to reduce a risk assessment's susceptibility to surprises, it is therefore necessary to narrow the knowledge gap between *C*′ in (2) and *C* in (1) as much as possible, while acknowledging that it can never be entirely closed. This is done by expanding the knowledge landscape into the unknown, thereby enabling possibilities beyond those represented in presently available knowledge to be derived from it. As demonstrated in the preceding sections of this article, CKT helps achieve this by combining, partitioning, recombining, and reshaping knowledge elements that represent established facts and decidable propositions. It is a process for generating novel conceptual possibilities that represent unknowns and undecidable propositions, which are then transformed into new knowledge elements in the form of established facts and decidable propositions through the four design operators. This expands a risk assessment's possibility space into the presently unknown and captures a broader range of possibilities within it. In this way, CKT aids the operationalization of the extended (*C*, *U*) risk‐assessment framework by narrowing the knowledge gap it highlights, from whence surprises arise. This may help in increasing the likelihood that what would otherwise be surprises are included in a risk assessment.

However, applying CKT to risk assessment in this way requires some adjustments and caveats. First, in CKT as applied to innovation, the decided status of an object in *K*‐space is deemed settled. It is an established fact. In contrast, in risk analysis, much knowledge is deemed defeasible, making the status of an element in *K*‐space provisional. In a risk context, *K*‐space may therefore be better understood as a time‐indexed knowledge base—*K*(*t*)—whose contents can be revised and expanded.^5^ This may be particularly important in open systems, where a key source of surprise is that something previously regarded as true turns out to be false. The proposition that the population of Iran would rise up and topple its remaining government once its leadership was decapitated may have been deemed true by those with decision‐making power in the United States in the lead‐up to the war in Iran (Derbyshire 2026). However, it has turned out to be false.

Second, we should be careful not to directly equate *K*‐space and *C*‐space with *K* and UK in the extended (*C*, *U*) risk‐assessment framework. First, for the reason stated above, the defeasibility of risk‐related knowledge, which means that the knowledge elements in *K*‐space must always be considered provisional. Second, because what are initially unknowns in *C*‐space—even after many partitions into a branching array of possibilities that stretches far from the disjunction from *K*‐space from whence it came—are forever only partially and not entirely unknown. There is still a tether connecting these novel possibilities to knowledge, not least because extending the array farther from its origins is an iterative process that involves transferring knowledge back into *K*‐space. At any one moment, both *K*‐space and *C*‐space therefore combine elements of knowledge and unknowledge and relate to both *K* and UK in the extended (*C*, *U*) risk‐assessment framework.

This combination of knowledge and unknowledge is reflected in a risk context in that even an extreme event that might be considered genuinely novel, such as a nuclear war, may have had historical precedents in the form of several near misses over the years (Alfano 2026). Relatedly, even what are presently unknown unknowns will have at least some basis in present knowledge (Derbyshire and Aven 2025), even if it is easier to see in hindsight than in foresight. Therefore, *K*‐space and *C*‐space are perhaps less distinctive from each other in a risk context than in an innovation context. The status of knowledge and unknowledge is far more fluid when it comes to risk.

To change tack, however, risk analysts have long used a variety of methods and concepts to expand a risk assessment's possibility space into the unknown. This expansion is not a new concept. How is the outlined CKT‐informed approach for expanding a possibility space into the unknown similar to or different from these other methods and concepts? In a final section, we briefly consider this question by discussing a few methods and concepts that have notable similarities to, and differences from, CKT.

## 5 Other Concepts and Methods Related to Expanding a Possibility Space Into the Unknown

### 5.1 Combining Common and Novel Causes to Anticipate Dragon Kings

A metaphorical menagerie has been employed to discuss the extreme and surprising events that the expansion of a possibility space is designed to capture. Some of the more exotic members of this menagerie have been discussed in Glette‐Iversen and Aven (2021), including the concept of “dragon kings” (DK), associated with Didier Sornette (Sornette and Ouillon 2012; Wheatley et al. 2017). The credit crunch can be considered a DK.

Through positive feedback and second‐order effects, an initially small event can develop into a runaway disaster that causes disproportionately more damage than other events of the same type (Wheatley et al. 2017). The term DK refers to just such events. They lie outside the distribution (usually a power law) that describes their commoner, more mundane cousins (Wheatley et al. 2017). DK is a double metaphor signaling an event that is both extremely large in size or impact (a “king”) and unique in origin (a “dragon”) compared with other events in the same system (Glette‐Iversen and Aven 2021).

The term “king” was coined (Laherrère and Sornette 1998) to describe these extreme outliers because the fortunes of kings far exceed those of the general population. DKs extend beyond what would be predicted by the power‐law distributions (e.g., the Pareto distribution) often used to model systems with extreme values. Traditionally, a king's wealth is off even that scale; it is a super‐extreme outlier. The term “dragon” was later added in recognition of the extraordinary characteristics of these events (Glette‐Iversen and Aven 2021). The phrase “here be dragons” refers to unknown territory on ancient maps (Glette‐Iversen and Aven 2021).

A DK grows large because it has novel causes that do not apply to its more mundane cousins, whose scale lies within and can be predicted by an appropriate power law (Wheatley et al. 2017). As the earlier discussion showed, the credit crunch combined many of the causal factors associated with previous financial crises, but its extremity extended far beyond that of most of them due to novel aspects, including increasing interconnectedness among banks and greater opacity in the financial system, as driven by the proliferation of complex financial instruments. Because CKT generates and transforms novel possibilities into established facts and decidable propositions, it can help anticipate DKs.

### 5.2 Extreme Value Theory: Uncovering an Unknown in the Form of a Hidden Maxima

The concept of a DK is different from but not greatly dissimilar to that of another member of the metaphorical menagerie of the extreme: the black swan (Taleb 2007). The two may have differing degrees of predictability, with the former being more predictable than the latter. Relatedly, a DK may be more representative of a known unknown than a black swan, which may be more of an unknown unknown. The degree to which one is more knowable in advance than the other is an interesting topic for another paper. However, we do not dwell specifically on this matter here. We refer any reader interested in unpicking the similarities and differences between DKs and black swans to Glette‐Iversen and Aven (2021) as a useful starting point.

More worthy of discussion here is Taleb's approach to identifying the possibility for extreme phenomena and unknowns. There is a deep skepticism towards forecasting and prediction, especially based on causal analysis, in Taleb's (2001, 2007, 2012) famous trilogy. Nevertheless, although he may eschew the attempt to predict any one instance of some classes of extreme event, Taleb *does* consider it possible to gain an inkling of the full potential extremity of a given event over time. Specifically, it is possible to gain an impression of an unknown in the form of a probability distribution's “hidden maxima” (Taleb 2020), which can be characterized as the “true” largest scale event of that type over infinite time. It contrasts with the in‐sample maxima, which might be misleading, as there is no guarantee that empirically observed past outturns are representative of the future's potential extremity.

In his short paper *What you see and what you don't see*, Taleb (2020) draws on Extreme Value Theory to discuss how every moment of an empirically derived probability distribution has a hidden equivalent: there is a hidden mean, maxima, variance, kurtosis, etc. The method Taleb (2020) sets out for identifying the hidden maxima enables calculation of the extent to which one might be wrong if one assumes that empirically derived past outturns accurately represent the true potential future extremity. As Taleb (2020, 2) describes it, the approach is useful for understanding the “properties of the unseen” in environments where past data provide insufficient evidence. It is, therefore, highly relevant to expanding the possibility space into the unknown.

However, the method does not help identify the causes of the potential extreme and says little about its potential timing. It only provides an indication of its full potential scale. For example, the method could indicate what to expect regarding the potential scale of a future financial crash. Yet this does not help anticipate the rhyming couplet that is the next instance, or when and how it might occur. For that, there may need to be some inkling regarding the novel aspects underlying its causal mechanism. Taleb's approach may therefore be of limited use to a regulator trying to anticipate and avert the specific occurrence of an extreme event, if not to one trying to make a system antifragile in the face of their possibility (Derbyshire and Wright 2014).

### 5.3 Robust Decision‐Making

The computational tool known as Robust Decision‐Making has been widely discussed in this journal (Hall et al. 2012; Lempert and Turner 2021; Reis and Shortridge 2020; Shortridge and Guikema 2016). It is explicitly designed to expand the possibility space into the unknown. RDM rejects simplistic attempts at optimization, or predicting the most likely future, or at estimating the probabilities of alternative outcomes. RDM stress‐tests alternative strategies across a very large number of scenarios and looks for options that perform satisfactorily across all or most of them, leading to a robust form of optimization. In so doing, it massively expands the possibility space beyond what a probabilistic approach based on evidence would yield. By running computer simulations across thousands or millions of plausible futures, RDM expands a possibility space to incorporate what were previously unknowns by exposing unexpected failure points and hidden trade‐offs that might otherwise have been overlooked. The CKT‐informed approach described herein might complement RDM by providing a framework for the stakeholder analyses that frame the analysis early in the RDM process.

### 5.4 The Baconian Scenario Algorithm

Considering power laws and super‐extreme outliers (as in a DK) and employing Extreme Value Theory to identify hidden maxima are both statistical means for identifying potential extremes and expanding the possibility space into the unknown. Yet, Dow et al. (2009) were earlier noted as suggesting that one reason for the failure to anticipate the credit crunch was mainstream economics’ excessive emphasis on mathematical modeling (Dow et al. 2009). Granted, the equilibrium‐based mathematical modeling at the center of mainstream economics and the statistical modeling of DKs and black swans is not the same thing. Nevertheless, the field of risk analysis also employs other, more qualitative and scenario‐based methods to anticipate extremes and surprises (Derbyshire et al. 2025). Indeed, RDM, as discussed above, is one example of a scenario‐based approach—albeit a more quantitative one that has qualitative aspects. We might consider more qualitative, scenario‐based methods more useful if the reason surprises tend not to be anticipated is—as in the cases of the credit crunch and 9/11—a failure of imagination. Through scenario analyses, human imagination and abductive reasoning might be applied, which could be crucial for anticipating such surprises.

Feduzi and Runde's (2014) Baconian scenario algorithm transforms unknowns into knowns. Its similarity to the CKT‐informed approach outlined herein comes immediately into focus when considering that it is a process of variation and elimination, in which extreme possibilities are considered one by one and either incorporated or eliminated until the bounds of plausible extremity have been reached. This similarity is also evident in the Baconian scenario algorithm generating unknowns as novel possibilities grounded in presently available knowledge.

As described in Derbyshire (2020), the algorithm starts from any base‐point state within an existing personal space of known possibilities that enjoys at least as much evidential support as any other. This becomes the jumping‐off point from which alternative states of the world are generated and tested for inclusion in, or elimination from, the possibility space. The decision‐maker imagines a state of the world, regardless of how unlikely it is in probabilistic terms, that is consistent with, yet not currently supported by, her personal knowledge set—one which, furthermore, if it were deemed part of the possibility space, would lie as far as possible from the base‐point state on either the negative or positive side of favorability in terms of its consequences and impact (it does not matter which, as the algorithm alternates).

The decision‐maker then seeks additional evidential support for the plausibility of this imagined state of the world that, if it were found, would lend support for it at least as high as that enjoyed by the base‐point state. This involves, among other things, considering its causal logic but could also draw on models, theories, mathematical propositions, engineering capabilities, expert knowledge, and analogies. The evidence acquired might lead to the inclusion or rejection of the newly considered state from the possibility space, the elimination of the base‐point state and other members of the original space, or the emergence of entirely new states of the world not previously considered (Derbyshire 2020).

Once the new state has been included in or excluded from the possibility space, as appropriate, the algorithm continues by imagining and considering a further state that is as extreme as possible in the same direction of favorability for inclusion or exclusion. The algorithm has a stopping mechanism. The scenario process continues until either a newly considered state receives such strong support that it supplants the current base‐point state on that side of the favorability scale, or imaginable states consistent with the current stock of evidence are exhausted. At this point, the process switches to the other side of the favorability scale. It is then conducted in the same way for that side. The process alternates between sides and stops when all imaginable new states have been considered and incorporated into the possibility space, or eliminated as possibilities, and the possibility space's bounds are therefore known (Derbyshire 2020).

There is quite a bit of crossover here with the outlined CKT‐informed approach. Yet, we might suggest, the Baconian scenario algorithm rests on a strong presumption of imaginative capacity. The process starts by imagining a new state of the world that is distant from the base point supported by presently available knowledge. There is, therefore, a question about the assessor's ability to escape the well‐known anchoring effect. According to Tversky and Kahneman (1974), the anchoring effect is the disproportionate influence of an initial value on judgment (Furnham and Boo 2011). If, in the Baconian scenario algorithm, the assessor starts from a base point that is as well supported as any other by available evidence, will this starting point anchor her ability to generate a novel possibility as far from that point as possible? On the other hand, it is worth noting that, once the decision‐maker has moved away from that base point, the approach has a built‐in mechanism designed to prevent confirmation bias: The scenario team must prove the newly considered scenario's possibility, rather than disprove it, which might be done all too glibly.

By contrast, in the CKT‐informed approach we have outlined, a branching array of possibilities is generated through what we might call, to borrow a term from the innovation literature (Corazza 2023; Kauffman 2000, 2014), movement into the “adjacent possible.” The initial disjunction from *K*‐space to *C*‐space is a nearby, incremental variation on a known possibility, not a distant one. This is then expanded in *C*‐space through partitions that are themselves a matter of incremental variations into the adjacent possible. The point is that the requirements placed on the assessor's imagination are not too onerous because there is no requirement to leap into the deep unknown by conjuring a novel possibility that is very distant from that which is the base starting point, and which has a strong foundation in presently available knowledge, as in the Baconian scenario algorithm. In CKT, expansion into the deep unknown is incremental and iterative; it does not require a large cognitive leap, which humans may struggle with due to the anchoring effect. In the CKT‐informed approach, expansion of a possibility space is based on and emergent from, but not constrained by, incremental variations of possibilities strongly supported by presently available knowledge.

## 6 Conclusion

To persuade policymakers to make risk‐reducing investments, the novel possibilities considered in a risk assessment cannot be mere guesses or figments of the imagination. They must be tethered to, but not entirely dependent on, presently available knowledge. Fortunately, there are very few entirely unprecedented events and outcomes. Even in open systems, much of what happens is a variation on a known theme. A risk assessor can therefore generate novel possibilities by expanding the possibility space outwards from the starting point of established facts and decidable propositions. As we have demonstrated, CKT provides a robust means to expand a risk assessment's possibility space into the unknown, such that the novel and previously unknown possibilities it considers are tethered to available knowledge.

That there is little (entirely) new under the sun is particularly evident in the case of financial crises, which tend to take one of several known forms, but with novel aspects in each instance. The question the Queen asked at the London School of Economics in 2008 was therefore a reasonable one: Why did nobody notice the credit crunch coming? After all, many of its features were similar to those of previous financial crises. Moreover, the answer—that it was a collective failure of the imagination—was also a reasonable one. But what that answer did not convey was the fact that acts of imagination are composed of something rather than nothing. Rather than mere pipedreams divorced from reality or evidence, acts of imagination would tend to be grounded in available knowledge.

Anticipating highly impactful surprises is, therefore, not a matter of making a lucky, imaginative stab in the absolute dark. There is light shining into the darkness from available knowledge. But instead of looking for a lost set of keys under a streetlight because that is where the light is, one must use the light to expand out into the surrounding darkness. Within CKT, novel possibilities are derived from the analysis of established facts and decidable propositions, thereby grounding them in knowledge. Yet, at the same time, room is left for abductive reasoning, imagination, and intuition, which come to the fore in expanding outwards into the unknown from the starting point of these underpinning facts and established propositions. In this way, the domains of knowledge and unknowledge are simultaneously expanded and integrated, generating a more comprehensive possibility space. This helps narrow the knowledge gap and anticipate what would otherwise be surprises when conducting risk assessments.

## Conflicts of Interest

The author declares no conflicts of interest.

## Data Availability

Data sharing is not applicable to this article as no datasets were generated or analyzed during the current study.

## Endnotes

1 The terms “credit crunch” and the “Great Financial Crash” are alternatively used to describe the financial crisis of 2007/2008. We refer to it herein as the credit crunch.

2 Two consecutive lines of a poem that end with words that rhyme with each other are called a “couplet.”

3 Undecidability is an established concept in number theory and computing science. See, for example, the undecidability inherent in Turing's (1936) “halting problem” (Cox 2020; Hamkins and Nenu 2026; Hatchuel and Weil 2009).

4 It should not be assumed that this testing and experimentation is necessarily empirical or experimental in the strictest sense of the word. Especially in a risk context, which we come to shortly, but also in the innovation context, *K*‐space can contain models, theories, mathematical propositions, engineering capabilities, expert knowledge, and analogies for which direct empirical evidence may be limited.

5 Gratitude must be given to an anonymous reviewer for this suggestion.
